# Cordycepin reduces weight through regulating gut microbiota in high-fat diet-induced obese rats

**DOI:** 10.1186/s12944-018-0910-6

**Published:** 2018-12-06

**Authors:** Yanan An, Yan Li, Xueyan Wang, Zhaobin Chen, Hongyue Xu, Lingyu Wu, Shulin Li, Chao Wang, Wenjing Luan, Xuefei Wang, Mingyuan Liu, Xudong Tang, Lu Yu

**Affiliations:** 1 Key Lab for New Drugs Research of TCM in Shenzhen, Research Institute of Tsinghua University in Shenzhen, Shenzhen, 518057 China; 2 Key Laboratory for Zoonosis Research, Ministry of Education, Institute of Zoonosis, College of Veterinary Medicine Jilin University, Changchun, 130062 China; 3 College of Life Science and Technology, Heilongjiang Bayi Agricultural University, Daqing, 163000 China; 4 West China School of Public Health, Sichuan University, Chengdu, 610041 Sichuan China; 5 Shenzhen Nanshan Center for Disease Control and prevention, Shenzhen, 518054 Guangdong China; 6 Jiangsu Co-innovation Center for Prevention and Control of Important Animal Infectious Diseases and Zoonosis, Yangzhou, 225009 China

**Keywords:** Cordycepin, Gut microbiota, Obesity, Body weight, Fat

## Abstract

**Background:**

An increasing number of studies have shown that obesity is the key etiological agent of cardiovascular diseases, nonalcoholic fatty liver disease, type 2 diabetes and several kinds of cancer and that gut microbiota change was one of the reasons suffering from obesity. At present, the gut microbiota has gained increased attention as a potential energy metabolism organ. Our recent study reported that cordycepin, a major bioactive component separated from Cordyceps militaris, prevented body weight gain in mice fed a high-fat diet directly acting to adipocytes, however, the effect of cordycepin regulating gut microbiota keeps unknown.

**Methods:**

In this research, we synthesized cordycepin (3-deoxyadenosine) by chemical methods and verified that cordycepin reduces body weight gain and fat accumulation around the epididymis and the kidneys of rats fed a high-fat diet. Furthermore, we used high-throughput sequencing on a MiSeq Illumina platform to test the species of intestinal bacteria in high-fat-diet-induced obese rats.

**Results:**

We found that cordycepin modifies the relative abundance of intestinal bacteria in high-fat-diet-induced obese rats. However, cordycepin did not alter the variety of bacteria in the intestine. Cordycepin treatment dramatically reversed the relative abundance of two dominant bacterial phyla (Bacteroidetes and Firmicutes) in the high-fat-diet-induced obese rats, resulting in abundance similar to that of the chow diet group.

**Conclusion:**

Our study suggests that cordycepin can reduce body weight and microbiome done by cordycepin seems be a result among its mechanisms of obesity reduction.

## Background

Obesity is an important issue in public health and has attracted increasing public attention around the world. Obesity can cause a variety of diseases, such as type 2 diabetes [1], nonalcoholic fatty liver disease, cardiovascular system diseases, insulin resistance, sleep apnea, osteoarthritis, cancer, asthma, and gall-bladder disease [2, 3]. The WHO has reported that 3.4 million adults die due to being obese or overweight. Obesity is mainly caused by excessive energy intake and sedentary lifestyle [4], which causes an imbalance in energy metabolism by reducing energy expenditure, leading to lipid accumulation in different tissues [5]. Therefore, it is particularly vital to protect people from obesity. It has been reported that obesity is closely related to chronic, low-grade inflammation and an alteration in intestinal flora. In addition, obese subjects exhibit systemic, chronic inflammation and a high level of serum endotoxins (lipopolysaccharides) that are associated with gut barrier dysfunction [6]. Until now, various mechanisms are reported to have relevance to obesity, including the changes of the locomotor activity, appetite through the pituitary or the hypothalamus in brain, the thermogenesis through brown adipose tissue, and lipogenesis in white adipose tissue, *etc* [7–10]. Especially, growing evidence indicates that the alteration of gut microbiota composition is linked to obesity and metabolism-associated diseases [11, 12]. The intestinal flora is made up of numerous bacteria that contribute to alimentation and the regulation of energy [13].

There are millions of microbes in the host intestine, such as bacteria, eukaryotes, and archaea, with bacteria being the most predominant. The colon is the last part of the digestive system and is full of gut microbes; the bacteria concentration is approximately 109–1012 CFU/mL, containing at least 1000 different species [14]. In the adult gut, approximately 90% of bacterial species are from the Firmicutes and Bacteroidetes phyla [15]. The Firmicutes phylum contains gram-positive bacteria from > 200 various genera, including Clostridium, Catenibacterium, Eubacterium, Lactobacillus, Faecalibacterium, Ruminococcus, Roseburia, Dorea, and Veillonella [14]. The Bacteroidetes phylum, which contains approximately 20 genera of gram-negative bacteria, such as Bacteroides, Prevotella, Tannerella and Odoribacter, is the second most widespread bacterial phylum. Other less abundant but common phyla of the intestinal flora include Actinobacteria, Verrucomicrobia and Proteobacteria [14]. The gut microbiota plays important roles in physiology and metabolism [16] by extracting energy from indigestible dietary compounds, mediating immunity and synthesizing vitamins. However, more and more research indicates that a change in gut microbiota is closely related to a variety of diseases. Abnormal changes in the gut flora usually affect host health by inducing an immune response [14, 17]. Interestingly, numerous studies have demonstrated the role of gut bacteria in obesity and metabolic dysfunction. In fact, some researchers have demonstrated a relationship between gut microbiota and the abundance of two bacterial phyla, Bacteroidetes and Firmicutes [4]. The first evidence of a change in gut microbiota composition in response to an obese phenotype was shown in genetically obese ob/ob mice; these mice displayed fewer Bacteroidetes and more Firmicutes bacteria [18]. The idea of an obesogenic gut microbial population emerged when the same authors discovered that the obese phenotype could be transmitted by gut microbiota transplantation in mice [19]. The increase in Firmicutes is related to an increase in enzymes that can disintegrate polysaccharides from food and produce short chain fatty acids (SCFA) [18].

It has been reported that butyrate and propionate could be anti-obesogenic and that acetate is mainly obesogenic. While butyrate is mainly produced by Firmicutes (the most important belonging to Clostridium clusters IV and XIVa, including Eubacterium rectale, Faecalibacterium prausnitzii, and Rosuberia intestinalis) and plays a significant role in maintaining host intestinal health [20], acetate and propionate are mainly produced by the phylum Bacteroidetes [18, 21, 22]. The gut microbiota is a potential therapeutic target for metabolic diseases. Although dietary interventions can normalize the composition of the gut microbiota in overweight and obese subjects, more targeted approaches are needed [23].

Cordycepin (CCS), namely, 3-deoxyadenosine, is a major bioactive component separated from Cordyceps militaris. Cumulative information has shown that cordycepin possesses multiple biological functions, such as antiviral, anti-inflammatory, anti-oxidant, anti-tumor, pro-apoptotic, anti-thrombotic activities [24, 25]. The others and our recent studies have demonstrated that cordycepin, a major bioactive component separated from *C. militaris*, prevents body weight gain in mice fed high-fat diets (HFD) [26, 27]. And we further found that cordycepin modulates body weight by reducing prolactin via an adenosine A1 receptor [26], or cordyceptin treatment affects pre-adipocytes differentiation, adipocytes growth and degradation in vitro and in vivo (our unpublished data). However, no data have shown that cordycepin reduces obesity by regulation of gut microbiota. In this study, we demonstrated that cordycepin can reduce high-fat-diet-induced obesity by regulating gut microbiota.

## Methods

### Animal experiments

All the animal experiments were performed in accordance with the Animal Welfare and Research Ethics Committee at Jilin University (no. IZ-2009-008). The protocols were reviewed and approved by the committee. Male SD rats with initial body weights of 180 ± 20 g were purchased from the Experimental Animal Centre of Jilin University in this study. All endeavors were made to reduce harm to rats. The temperature was 25 ± 1 °C, and relative humidity was 40–80%. Both the high-fat diet (60% kcal, D12492) and normal chow diet for rats (10% Kcal, D12450K) were provided by Research Diets of the USA. Cordycepin (purity > 99%) was provided by the Academy of Tsinghua University, Shenzhen China.

After seven days of acclimation, 30 rats were randomly divided into three groups based on their body weight, with no significant differences among the three groups: (1) fed a normal diet (NFD) group (*n* = 10); (2) fed a 60% high-fat diet (HFD) group (*n* = 10); and (3) the HFD + CCS group (n = 10), administered cordycepin (50 mg/kg) by gavage once a day for 4 weeks simultaneously with the 60% high-fat diet. Cordycepin was dissolved in distilled water before intragastric administration. The rats in the NFD group and the HFD group were given the same volume of distilled water, exactly the same as the cordycepin-treated group.

During the experiment, body mass was monitored weekly, and the administered dose was adjusted accordingly. In the end, fasting body mass was measured after precisely 12 h without food, and then, all rats were euthanized. Tissues, including perirenal fat and epididymal fat, were excised and weighed. The adiposity index was then calculated according to the following formula [28]: (epididymal fat weight + perirenal fat weight) × 100/body weight. In addition, the contents of the intestine were collected under aseptic conditions and immediately frozen in liquid nitrogen for 16S rDNA sequencing.

### Sequencing of the 16S rDNA V3 and V4 regions of intestinal microbiota

A Micro Elute Genomic DNA Kit (D3096–01, Omega, Inc., USA) was used for extracting total genomic DNA from intestinal microbiota. The specific extraction method was performed in accordance with the manufacturer’s directions. Then, 16S rDNA V3 and V4 double variable regions from each sample were amplified via polymerase chain reaction (PCR) with Primers 319F (ACTCCTACGGGAGGCAGCAG) and 806R (GGACTACHVGGGTWTCTAAT); this region (V3-V4) was used for PCR because there is great diversity among bacteria phyla. Amplification was performed with the DNA template (50 ng) in a 25 μL system for 25–35 cycles with Phusion DNA Polymerase. Extraction of the total genomic DNA from the intestinal microbiota and amplification of the pyrosequencing were performed on an Illumina MiSeq platform at LC-Bio Technology Company, Hangzhou, Zhejiang Province, China.

### Bioinformatics

The overlapped Illumina MiSeq data, 2 × 300 bp, were paired-end merged and spliced to tags. Then, the tags were filtered by QIIME (Quantitative Insights into Microbial Ecology) quality filters. Put simply, each read that was counted as effective data was reserved, and those that did not meet all of the requirements were excluded from subsequent analysis.

Then, the sequences were grouped into operational taxonomy units (OTU) at 97% similarity. Basically, there was less than 3% sequence dissimilarity in all reads of the same OTU. The RPD (Ribosomal Database Project) was applied to classify the OTU sequence and identify the bacterial species.

### Statistical analysis

All data analyses in this study were subjected to one-way ANOVA using SPSS Statistic 19.0 (IBM, USA). A less than 5% difference between individual means was deemed as significant.

## Results

### Design and synthesis of cordycepin

**Fig. 1:**
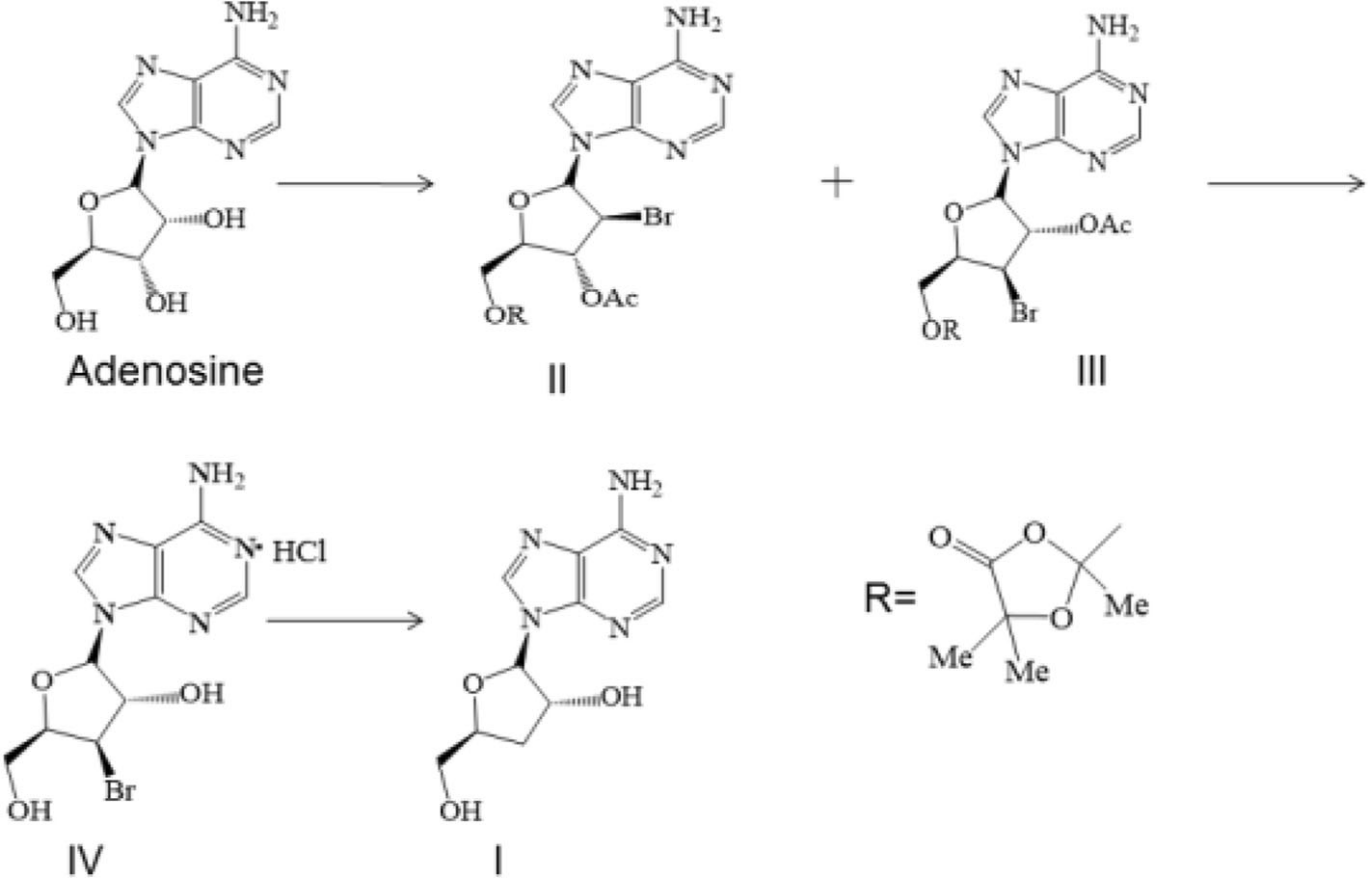
Synthetic route and chemical structure of cordycepin. (I) Cordycepin. (II, III, IV) intermediate products

In our study, cordycepin was synthesized by chemical methods. Adenosine, which is inexpensive, was used as the starting material and then selectively replaced by Mattock’s bromide for the bromination of adenosine 3′ hydroxyl groups, followed by hydrolysis to obtain 3′ bromo adenosine. Finally, debromination by catalyzed hydrogenation was performed to obtain the target product, cordycepin. The synthetic route and chemical structure of cordycepin are shown in (Fig. 1).

### Cordycepin reduced body weight and fat in high-fat-diet-induced obese rats

**Fig. 2:**
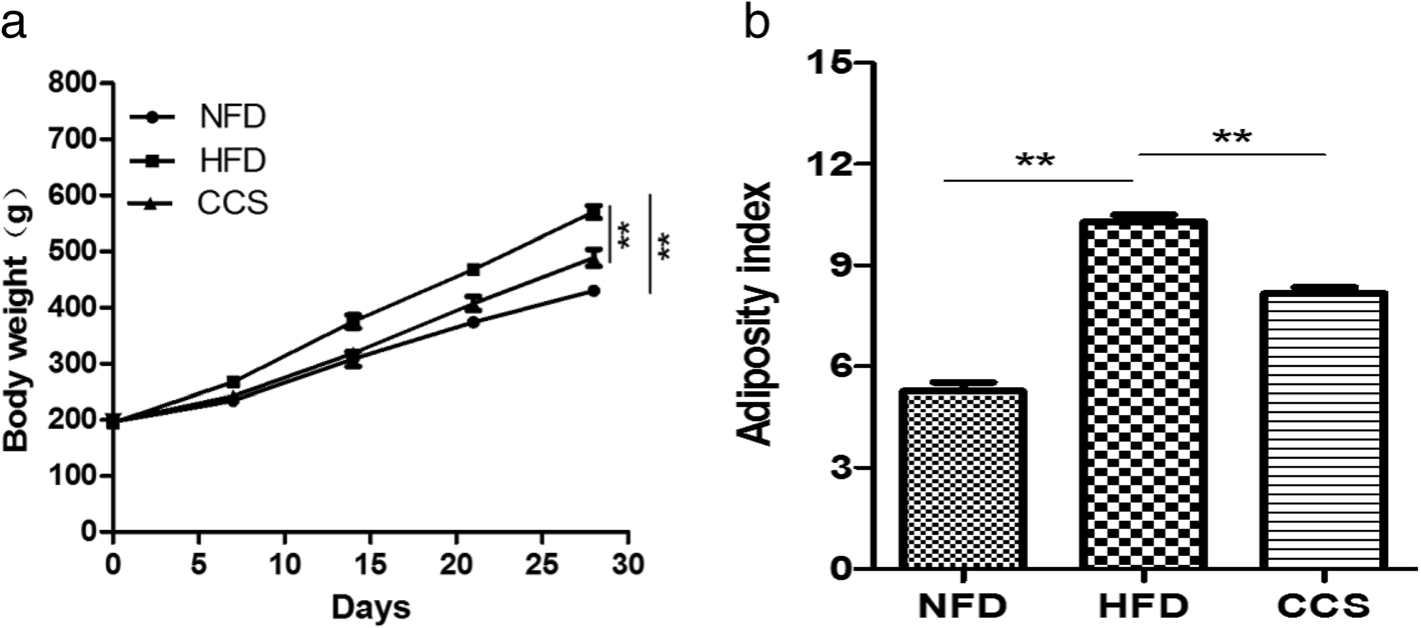
Anti-obesity effects of cordycepin in high-fat-diet-induced obese rats. (**a**) Body weight. (**b**) Adiposity index, calculated according to the following formula: 100 × (epididymal fat weight + perirenal fat weight)/body weight. Differences were assessed by one-way ANOVA, (*P < 0.05, **P < 0.01, ***P < 0.001). NFD means the rats were fed a normal diet; HFD meansthe rats were fed a high-fat diet; CCS means the rats were fed a high-fat diet and received cordycepin (50 mg/kg) for 4 weeks continuously

To investigate the effect of cordycepin on body weight and fat, we established the obese rat model by feeding rats a high-fat diet. Cordycepin (50 mg/kg/d) was orally administered when the 4-week high-fat diet began. Our results showed that obesity was significantly induced from week 2 to week 4 in rats that were fed a high-fat diet (Fig. 2a). Cordycepin significantly reduced this increase in body weight in high-fat-diet-induced obese rats (*P* < 0.01) (Fig. 2a) and inhibited the accumulation of body fat compared with the HFD group from week 2 to week 4 (*P* < 0.01) (Fig. 2b). In general, cordycepin could reduce body weight and fat in the high-fat-diet-induced obese rats.

### Alteration of gut microbiota structure by cordycepin in high-fat-diet-induced obese rats

**Fig. 3:**
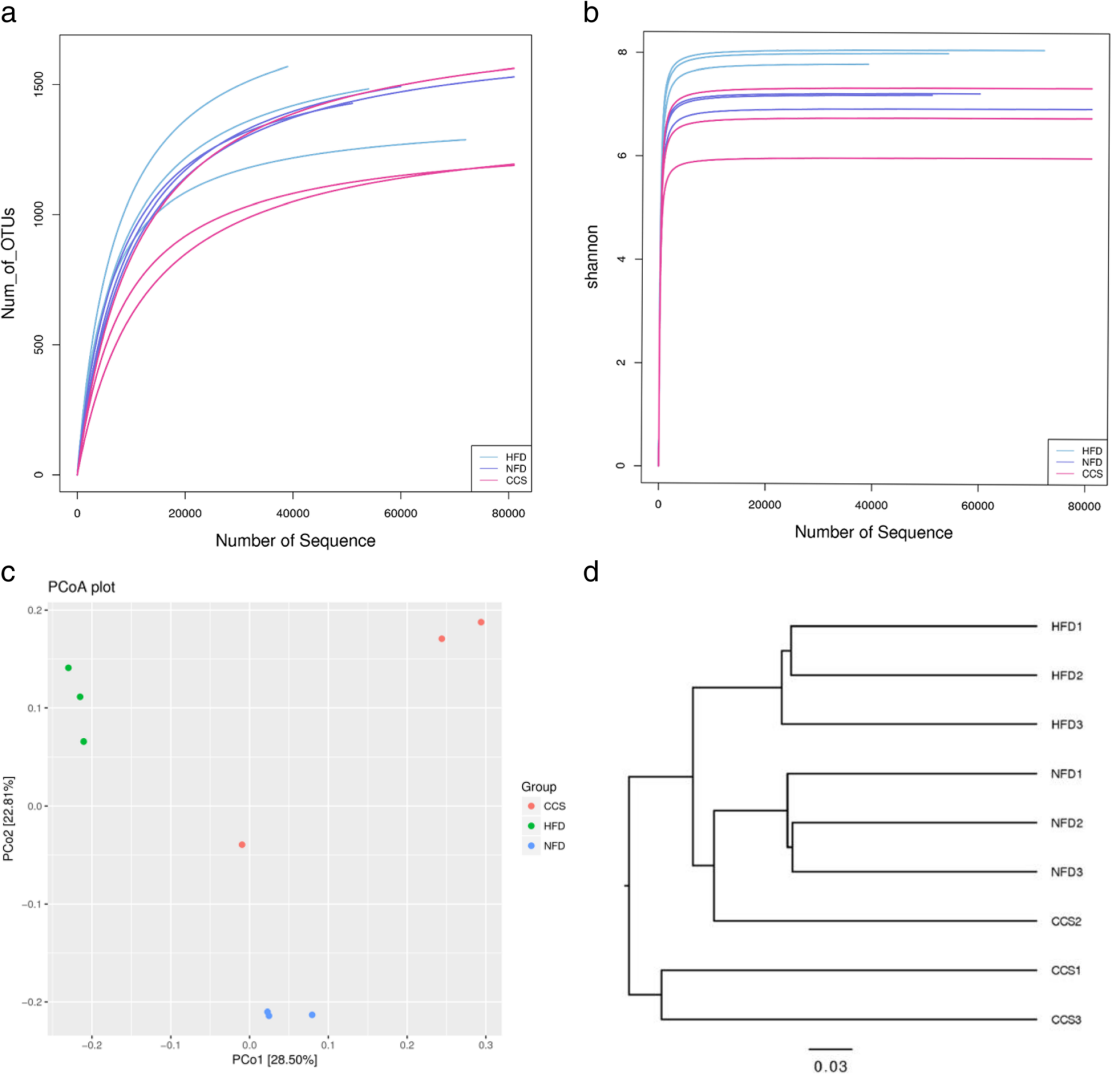
Diversity and richness of the gut microbiota in rats. (**a**) Displays the rarefaction curves of samples from the NFD group, HFD group and CCS group. (**b**) Displays the Shannon index of samples from the NFD group, HFD group and CCS group. (**c**) PCoA plot. Principal component analysis (PCA) scores plot of gut microbiota based on the relative abundance of OTUs. (**d**) Cluster analysis, unweighted uniFrac-OTU table. NFD means that the rats were fed a normal diet (*n* = 3), HFD meansthe rats were fed a high-fat diet (*n* = 3), and CCS meansthe rats were fed a high-fat diet and received cordycepin (50 mg/kg) (*n* = 3) for 4 weeks continuously

To assay the effect of cordycepin on the regulation of gut microbiota structure, high-throughput pyrosequencing was performed with an Illumina MiSeq platform to generate 820,838 high quality and valid sequences from 9 intestinal content samples from different groups. The NFD group produced 73,056 ± 20540sequences per sample, the HFD group produced 92,682 ± 23654sequences per sample, and the CCS group produced 107,874 ± 5608sequences per sample. The high-quality sequences were assigned into 3303 operational taxonomic units (OTUs) at 97% similarity. We found that although new species could be obtained by increasing the sequences, most of the intestinal microbiota diversity could be acquired with the current sequencing depth by Shannon and Rarefaction analysis (Fig. 3a, b). The Shannon diversity index and rarefaction OTU estimates were calculated by rarefying the sequencing depth among all the samples using a helper application. There were no significant differences in species abundance and diversity by analysis of Shannon, Simpson and Chao1 indices among the NFD, HFD and CCS groups.

UniFrac-based principal coordinates analysis (PCoA) showed obvious clustering of gut microbiota composition for each treated group. Multivariate analysis of variance of the PCoA matrix scores showed a statistically significant separation among the microbiota of the NFD group, HFD group and CCS group (Fig. 3c). However, when the cordycepin, NFD, and HFD groups were compared, significant separations were found (Fig. 3d).

It is noteworthy that species taxonomy proved that the gut microbiota composition was significantly changed in high-fat-diet-induced obese rats by administering cordycepin for 4 weeks. A total of 13 bacterial phyla were detected by 16S rDNA sequencing: Firmicutes, Bacteroidetes, Proteobacteria, Cyanobacteria, Spirochaetes, Tenericutes, Deferribacteres, Candidatus Sacchari bacteria, Actinobacteria, Elusimicrobia, Verrucomicrobia, Fusobacteria, and Lentisphaerae. Among these phyla, Firmicutes, Bacteroidetes, and Proteobacteria accounted for 98% of the total, and the percentage of all other phyla was 2%.

**Fig. 4:**
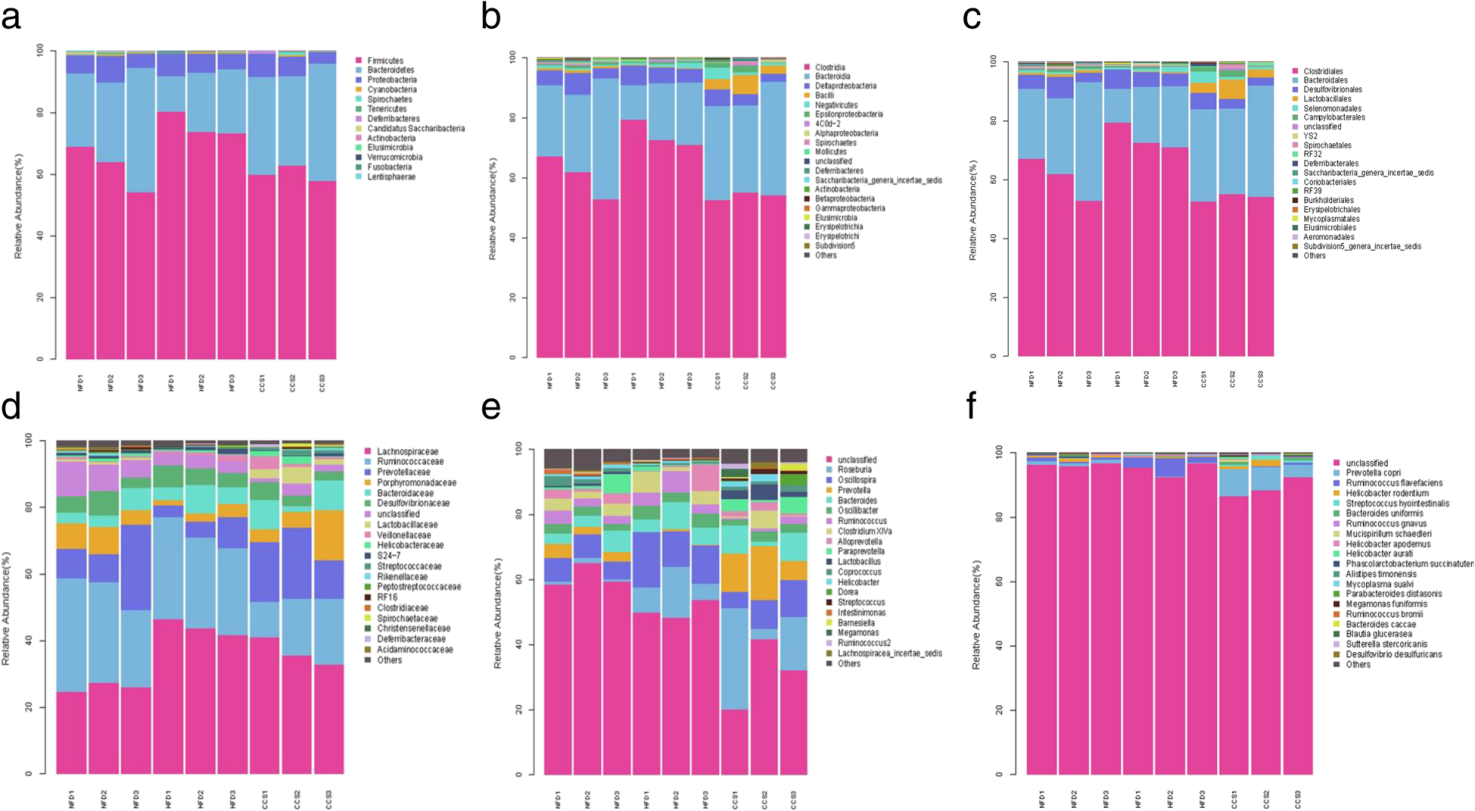
Composition analysis of gut microbiota at different taxonomic levels among all samples. (**a**) Phylum. (**b**) Class. (**c**) Order. (**d**) Family. (**e**) Genus. (**f**) Species. Only the top 20 most abundant OTUs are displayed. NFD means that the rats were fed a normal diet (*n* = 3), HFD meansthe rats were fed a high-fat diet (*n* = 3), and CCS means the rats were fed a high-fat diet and received cordycepin (50 mg/kg) (*n* = 3) for 4 weeks continuously

Gut microbiota was analyzed by 16S rDNA pyrosequencing at the levels of phylum, class, order, family, genus and species (Fig. 4). At the level of phylum, compared with the NFD group, the HFD group had a significant decrease in the phylum Bacteroidetes and are mark able increase in the phylum Firmicutes. However, compared with the HFD group, cordycepin treatment reduced the phylum Firmicutes and increased the phylum Bacteroidetes in the high-fat-diet-induced obese rats; these changes restored levels to that of the group fed a normal diet. However, there was no significant difference in the level of Proteobacteria among the three groups (Fig. 4a).

At the level of class, rats in the HFD group presented a significantly higher level of Clostridia, (a class of the Firmicutes phylum) as well as a significant reduction in Bacteroidia (a class of the Bacteroidetes phylum); the percentage of the two bacterial classes in the cordycepin-treated group was reverted to the level of the NFD group (NFD group as a reference) (Fig. 4b).

At the level of order, compared with the NFD group, the HFD group showed that Bacteroidales (an order of the Bacteroidiaclass, Bacteroidetes phylum) and Lactobacillales (an order of the Bacillales, Firmicutes phylum) were significantly reduced, whereas Pasteurellales (an order of the Gamma proteobacteria class, Proteobacteria phylum), Bacillales (an order of the Bacilli, Firmicutes phylum), and Clostridiales (an order of the Clostridia, Firmicutes phylum) were significantly increased. The above changes were reverted by cordycepin to a level similar to that of the NFD group (NFD group as a reference) (Fig. 4c).

At the level of family, rats in the HFD group presented significantly higher levels of Veillonellaceae, Lachnospiraceae (a family of the Clostridiales order, Clostridia class, Firmicutes phylum), and Pasteurellaceae (a family of the Pasteurellales order, Gammaproteobacteria class, Proteobacteria phylum) and lower levels of Lactobacillaceae (a family of the Lactobacillales order, Bacilli class, Firmicutes phylum), Spirochaetaceae (a family of the Spirochaetales order, Spirochaetes class, Spirochaetes phylum), Eubacteriaceae (a family of the Clostridiales order, Clostridia class, Firmicutes phylum), and Prevotellaceae (a family of the Bacteroidales order, Bacteroidia class, Bacteroidetes phylum). Moreover, cordycepin prevented these changes in the high-fat-diet-induced obese rats to a level similar to that of the NFD group (Fig. 4d).

At the level of genus, compared with the NFD group, Lactobacillus (a genus of the Lactobacillaceae family, Lactobacillales order, Bacilli class, Firmicutes phylum), Clostridium XlVb (a genus of the Lachnospiraceae family, Clostridiales order, Clostridia class, Firmicutes phylum), Prevotella (a genus of the Prevotellaceae family, Bacteroidales order, Bacteroidia class, Bacteroidetes phylum), Anaerovibrio (a genus of the Veillonellaceae family, Selenomonadales order, Negativicutes class, Firmicutes phylum), Eubacterium (a genus of the Eubacteriaceae family, Clostridiales order, Clostridia class, Firmicutes phylum), Parabacteroides (a genus of the Porphyromonadaceae family, Bacteroidales order, Bacteroidia class, Bacteroidetes phylum) were significantly reduced and Clostridium IV, Oscillospira (a genus of Ruminococcaceae family, Clostridial order, Clostridia class, Firmicutes phylum) was significantly increased in the HFD group. In addition, cordycepin reduced these changes in the high-fat-diet-induced obese rats to a level similar to that of the NFD group (Fig. 4e).

At the level of species, compared with NFD group, Butyric monas synergistica (a species of the Butyric monas genus, Porphyromonadaceae family, Bacteroidales order, Bacteroidia class, Bacteroidetes phylum) and Ruminococcus flavefaciens (a species of the Ruminococcus genus, Ruminococcaceae family, Clostridiales order, Clostridia class, Firmicutes phylum) were significantly increased, whereas Prevotellacopri (a species of the Prevotella genus, Prevotellaceae family, Bacteroidales order, Bacteroidia class, Bacteroidetes phylum) was significantly decreased. Compared to the HFD group, the relative distributions of the three bacteriain the cordycepin-treated group were reverted to levels similar to those of the NFD group (Fig. 4e).

**Fig. 5:**
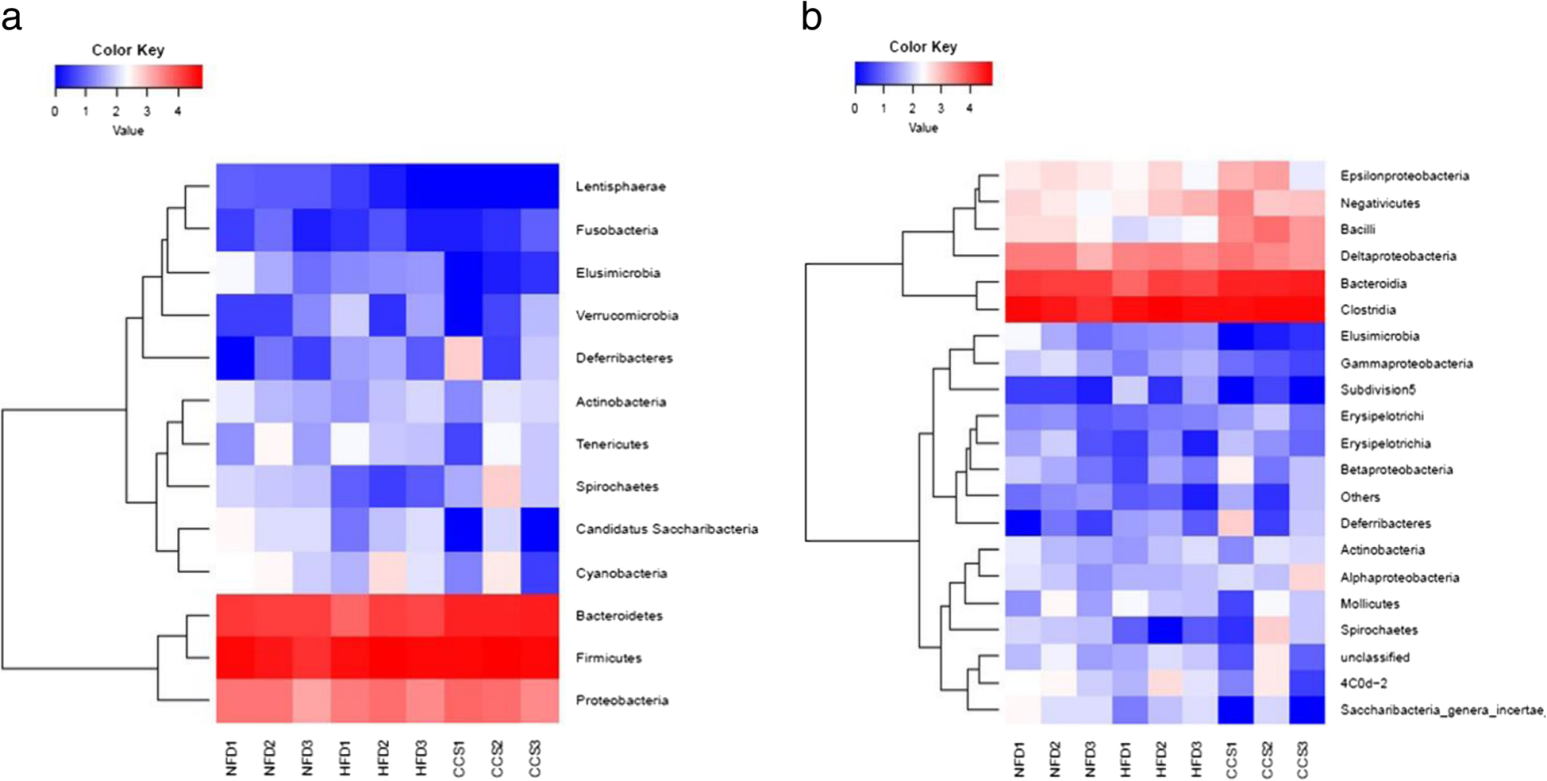
Heatmap analysis of gut microbiota changes from different treatments. (**a**) Phylum. (**b**) Class.The color intensity in each sample is normalized to represent its relative ratio in the three groups. A range of colors, from blue to red, indicates the relative values of microbiota (0–4). NFD means that the rats were fed a normal diet (*n* = 3), HFD meansthe rats were fed a high-fat diet (*n* = 3), and CCS meansthe rats were fed a high-fat diet andreceived cordycepin (50 mg/kg) (*n* = 3)for 4 weeks continuously

Generally, our results showed that rats in the HFD group exhibited a lower level of the Bacteroidetes phylum and a higher level of the Firmicutes phylum than those in the normal diet group. Furthermore, cordycepin reduced the percentage of the Firmicutes phylum and increased the percentage of the Bacteroidetes phylum in the high-fat-diet-induced obese rats. Some changes in phylum were mostly in conformity at downstream levels. For instance, the Clostridia class, Bacillales order, Clostridiales order, Veillonellaceae family, Lachnospiraceae family, Clostridium IV genus, Oscillospira genus, and Ruminococcus flavefaciens species, all belonging to the Firmicutes phylum, were significantly increased in the HFD group. Moreover, the Bacteroidia class, Bacteroidales order, Prevotellaceae family, Prevotella genus, Parabacteroides genus, and Prevotellacopri species, all belonging to the Bacteroidetes phylum, were significantly reduced in obese rats. However, there were some inconsistencies downstream of the level of phylum. For instance, Lactobacillales order, Clostridium XlVb order, Lactobacillaceae family, Eubacteriaceae family, Lactobacillus genus, Clostridium XlVb genus, Anaerovibrio genus, and Eu bacterium genus, which belong to the Firmicutes phylum, were significantly decreased. However, Butyric monas synergistica, belonging to the Bacteroidetes phylum, was dramatically reduced in ratsof the HFD group. The changes in the relative abundance of Firmicutes and Bacteroidetes at phylum and class levels can be seen in the heatmap (Fig. 5a, b).

## Discussion

Previous studies have reported that cordycepin extracted from *C. militaris* can reduce body weight gain in mice [27], and our recent result showed that cordycepin modulates body weight by reducing prolactin via an adenosine A1 receptor [26], or cordyceptin regulates body weight by inhibiting pre-adipocytes differentiation and adipocytes growth, and inducing adipocytes degradation (our unpublished data), these results suggested that cordycepin could directly act to adipocyte in terms of lipid metabolism. However, the ability of cordycepin to reduce body weight gain by modulating the distribution of gut microbiota in high-fat-diet-induced obese rats has not yet been investigated. In our study, we used adenosine to synthesize cordycepin by chemical methods and then verified that cordycepin has beneficial effects on reducing both body weight gain and fat accumulation around the epididymis and kidney, and we discovered that cordycepin can regulate the constitution of gut microbiota in HFD-induced obese rats.

Many previous studies have shown that obesity is closely related to the structure of intestinal microbiota. Higher amounts of Bacteroidetes in the gut microbiota are directly connected with a lean phenotype [29] and with obese individuals who lose weight; reports both in rats and in humans have demonstrated that the relative abundance of Bacteroidetes is reduced by a high-fat diet [30]. An increasing number of researchers have demonstrated that obesity is associated with changes in the relative abundance of Bacteroidetes and Firmicutes or in the ratio of Bacteroidetes to Firmicutes. Generally, there is an increase in Firmicutes and a decrease in Bacteroidetes in obese rats and humans [31, 32]. Similarly, our results display that the Firmicutes phylum was significantly increased and the Bacteroidetes phylum was significantly reduced in high-fat-diet-induced obese rats. Accordingly, administration of cordycepin prevented diet-induced obesity and shifted the relative abundance of gut microbiota in high-fat-diet-induced obese rats. Namely, there was less of the Firmicutes phylum and more of the Bacteroidetes phylum in cordycepin-treated high-fat-diet-induced obese rats. According to Turnbaugh et al., a low-energy diet increased the percentage of Bacteroidetes in the gut microbiota [31]. In contrast, there was more Firmicutes in rats fed a high-fat diet [18]. Changes in gut microbiota may require dietary intervention [23]. Transplantation of microbiota harvested from conventionally raised mice into germ-free mice resulted in an increase in body weight and decrease in insulin sensitivity; this fact further supports the concept that body weight could be regulated by gut microbiota. Despite the controversy, studies consistently show an increase in Firmicutes and a decrease in Bacteroidetes in obese rats. Increased abundance of Firmicutes in the intestinal microbiota of obese patients has been suggested to increase the capacity to harvest energy from the diet, thus promoting more efficient absorption of calories and subsequent weight gain [19]. Moreover, compared with Bacteroidetes, a higher proportion of Firmicutes has been described in genetically or diet-induced obese mice. This increase in Firmicutes is connected to an increase in enzymes able to breakdown indigestible polysaccharides from the diet and produce short chain fatty acids (SCFA). Lactobacillus and Bifidobacterium are often used as probiotics [33], and previous studies have shown an increase in Lactobacillus and Bifidobacterium in obese individuals [34, 35]. However, Lecomte V et al. indicated that the abundance of these two species was negatively correlated with fat mass and body weight [19]. Our study showed less Lactobacillus in obese rats, and Lactobacillus was significantly increased in cordycepin-treated rats, which was similar to the normal diet group. In fact, the relationship between Lactobacillus and obesity could be dependent on the experimental model or Lactobacillus species [36]. Cordycepin treatment also displayed a positive impact on the Prevotella genus (Prevotellacopri species), which are well-known intestinal bacteria. Therefore, cordycepin might regulate relative metabolism by increasing probiotics. However, we found that Bifidobacterium was not changed among the three groups.

It was reported that there is an increase in Oscillibacter spp. in rats fed a high-fat diet and that these bacteria have a negative connection with the expression of intestinal tight junction proteins [37], which is similar to our results. In this study, cordycepin treatment showed a significant decrease in the Oscillospira genus compared with the HFD group, similar to chow-fed rats.

Bacteria belonging to Clostridium clusters XIVa, XVIII and IV, which lack virulence factors and prominent toxins, were found to modulate host fatty acid metabolism, induce Treg cell activity and attenuate colitis [38]. Moreover, the Eubacterium spp. induced by prebiotic oligosaccharides produced beneficial effects on animal hosts, highlighting the potential probiotic effect of this species. Our results showed that the Eubacterium genus and Clostridium XlVb increased significantly in rats treated with cordycepin, which was similar to the chow diet group. Previous research reported that Eubacterium and Clostridium XlVb could produce butyrate, which has an anti-obesity effect [20]. These results indicate that the effects of cordycepin on reducing body weight gain and fat accumulation might be at least partially due to an increase in the population of these beneficial species. Additionally, in our results, we found that the pathogen Pasteurellaceae, belonging to the Proteobacteria phylum [39], significantly increased in the HFD group, while Pasteurellaceae significantly decreased in cordycepin-treated rats. Moreover, there was a lower level of the Spirochaetaceae family (Spirochaetes phylum) in the obese rats but a higher level in the cordycepin-treated rats.

Obesity implies an imbalance between energy intake and expenditure, resulting in an excess of energy storage as adipose tissue. Moreover, many studies showing an increase in body weight and fat in germ-free mice after transplanting gut microbiota derived from wild as well as from obese-mice [31, 40], and this can be a proof that the gut microbiota of obese individuals is more efficient at extracting energy from the diet than the microbiota of lean individuals. And several gut microbiota related mechanisms can be explained the weight gain, such as the microbial fermentation of indigestible dietary polysaccharides into absorbable monosaccharides, and the generation of short-chain fatty acids (SCFAs) which are converted to more complex lipids in the liver, moreover, Firmicutes are major producers of the SCFA butyrate [41–43]. Our results showed an increase in the number of Firmicutes in the high-fat-diet-induced obese rats, while the cordycepin treatment group significantly reduced the number and proportion of Firmicutes, which is consistent with previous reports.

A growing number of studies have shown that the gut microbiome may influence brain activity and behaviors. For example, several preclinical studies have demonstrated that manipulation of the gut microbiota can alter emotional, nociceptive, and social behaviors [44], and produce region specific neurochemical brain changes [45]. According to previous reports that the gut microbiome has been associated with changes in brain microstructure and hormone secretion in obesity, by produces several neuroactive compounds including several in dole-containing metabolites and 5-HT, and distinct microbial brain signatures were able to differentiate obese from lean subjects [46, 47]. From this we can know that the gut microbiome is involved in the production of obesity in many aspects. In addition, in our results cordycepin shifted the relative abundance of gut microbiota (especially, less of the Firmicutes phylum and more of the Bacteroidetes phylum) in high-fat-diet-induced obese rats, and made it successfully lose weight. All of these results and papers confirmed that the gut microbiomemay be closely related to food intake, brain microstructural and hormone secretion.

Together, our results showed that cordycepin has a direct effect on the endocrine system and the conversion between fats, especially, the influence of intestinal flora on the fat by cordycepin found in this study is also a very important aspect. All these results we found indicated that the mechanisms of the effect of cordycepin on the fat could be complicated and mediated through multi-mechanisms, and these mechanisms may play a cross role on the the effect of obesity reduction by cordycepin, however, the relationship among these mechanisms remains to be further studied.

## Conclusions

In conclusion, our research suggests that regulating the constitution of gut microbiota might be one of mechanisms of obesity reduction by cordycepin and that these results provide a potential way to treat obesity.

## Abbreviations

CCS: Cordycepin
HFD: Fed a 60% high-fat diet
NFD: Fed a normal diet
OTUs: Operational taxonomic units
PCoA: UniFrac-based principal coordinates analysis
QIIME: Quantitative Insights into Microbial Ecology
SCFA: Short chain fatty acids
